# RhoA promotes epidermal stem cell proliferation via PKN1-cyclin D1 signaling

**DOI:** 10.1371/journal.pone.0172613

**Published:** 2017-02-21

**Authors:** Fan Wang, Rixing Zhan, Liang Chen, Xia Dai, Wenping Wang, Rui Guo, Xiaoge Li, Zhe Li, Liang Wang, Shupeng Huang, Jie Shen, Shirong Li, Chuan Cao

**Affiliations:** 1 Department of Plastic and Reconstructive Surgery, Southwestern Hospital, Third Military Medical University, Chongqing, China; 2 School of Nursing, Third Military Medical University, Chongqing, China; University of Texas at Austin Dell Medical School, UNITED STATES

## Abstract

**Objective:**

Epidermal stem cells (ESCs) play a critical role in wound healing, but the mechanism underlying ESC proliferation is not well defined. Here, we explore the effects of RhoA on ESC proliferation and the possible underlying mechanism.

**Methods:**

Human ESCs were enriched by rapid adhesion to collagen IV. RhoA^(+/+)^(G14V), RhoA^(-/-)^(T19N) and pGFP control plasmids were transfected into human ESCs. The effect of RhoA on cell proliferation was detected by cell proliferation and DNA synthesis assays. Induction of PKN1 activity by RhoA was determined by immunoblot analysis, and the effects of PKN1 on RhoA in terms of inducing cell proliferation and cyclin D1 expression were detected using specific siRNA targeting PKN1. The effects of U-46619 (a RhoA agonist) and C3 transferase (a RhoA antagonist) on ESC proliferation were observed in vivo.

**Results:**

RhoA had a positive effect on ESC proliferation, and PKN1 activity was up-regulated by the active RhoA mutant (G14V) and suppressed by RhoA T19N. Moreover, the ability of RhoA to promote ESC proliferation and DNA synthesis was interrupted by PKN1 siRNA. Additionally, cyclin D1 protein and mRNA expression levels were up-regulated by RhoA G14V, and these effects were inhibited by siRNA-mediated knock-down of PKN1. RhoA also promoted ESC proliferation via PKN in vivo.

**Conclusion:**

This study shows that the effect of RhoA on ESC proliferation is mediated by activation of the PKN1-cyclin D1 pathway in vitro, suggesting that RhoA may serve as a new therapeutic target for wound healing.

## Introduction

The skin is the largest organ of the body and the primary protective barrier against the environment [1]. The epidermis, consisting of keratinocytes with variable degrees of differentiation, is constantly maintained by self-renewing epidermal stem cells (ESCs) [2], skin-specific adult stem cells with a strong proliferative capacity. Following induction, ESCs differentiate into a variety of epidermal lineages to promote self-renewal and regeneration of the epidermis as well as wound healing [3]. Wound healing is a complex process mediated by various factors responsible for the regeneration and reorganization of damaged tissue into its normal architecture, and ESC differentiation, migration and proliferation are the basis of this process. We previously conducted work on ESC differentiation [4] and migration [5,6]; however, little is known regarding ESC proliferation.

The small GTPase RhoA, a member of the Rho GTPase family (RhoA, CDC42 and Rac), is activated by guanine nucleotide exchange factors (GEFs) [7]. Numerous biochemical, cell biological and physiological studies have demonstrated that RhoA tightly regulates actin-based structure formation [8], cell proliferation [9,10] and cell migration [11,12]. PKN (protein kinase N; also referred to as PRK, protein kinase C-related kinase) plays an important role in diverse functions, including regulation of the cell cycle [13], proliferation [14], migration [15] and apoptosis [16]. PKNs can be activated by binding to Rho and certain unsaturated fatty acids such as arachidonic acid [17]. Moreover, PKN is involved in transforming growth factor (TGF)-beta1-induced expression of smooth muscle marker genes after RhoA activation [18]. PKNs have been implicated in signal transduction as effectors of Rho, Rac and PI3K (phosphoinositide 3-kinase) [19].

At least three isoforms of PKN are present in mammals, termed PKN1, PKN2 and PKN3 [20]. Notably, PKN1 regulates endometrial cancer cell proliferation by modulating TGFβ and epidermal growth factor (EGF) dependence, promotes prostate cancer cell proliferation through WDR5 (WD repeat-containing protein 5) [21,22]. In particular, PKN1 plays a critical role in vascular wall remodeling and accelerates smooth muscle cell migration and proliferation linked to cyclin D1 [23]. Cyclin D1 plays a central role in regulating proliferation by linking the extracellular signaling environment to cell cycle progression [24,25]. Cyclin D1 expression is highly responsive to proliferative signals, including growth factor receptors, Ras, and their downstream effectors. However, the critical cyclin D1-mediated proliferation of ESCs has not been fully elucidated.

Our previous study suggested that RhoA was linked to ESC migration mediated by nitric oxide [6]. Therefore, in the present study, we employed cultured ESCs prepared from human foreskin and primarily focused on the effect of RhoA on ESC proliferation. The present study demonstrated that RhoA promotes ESC proliferation through PKN1-cyclin D1 signaling, suggesting that RhoA may be involving in wound healing.

## Materials and methods

### Isolation, culture and identification of primary human ESCs

Primary human ESCs (huESCs) were isolated from human neonatal foreskins using a modified method of rapid adhesion to collagen IV as previously described [26]. Human skin samples used for cell cultures were obtained from neonatal foreskins; the samples were de-identified and were to be discarded, which exempts the use of this tissue from requirements for informed consent according to 45CFR46. This exempt status for collection of discard skin without informed consent was confirmed by the Ethics Committee of Southwest Hospital, Chongqing, China. Briefly, foreskin was incubated overnight in 0.25% Dispase II (Roche, Basel, Switzerland) at 4°C. Then, separated epidermis was dissociated into single cells with 0.25% trypsin for 10 min at 37°C. The isolated cells were suspended in keratinocyte serum-free medium (K-SFM, Invitrogen, CA, USA) supplemented with 0.2 ng/ml EGF, 20 mg/ml bovine pituitary extract, 0.05 mM CaCl_2_ and 100 IU/ml streptomycin/penicillin. Then, 1×10^6^ cells were selected for 10 min at 37°C on 25-mm^2^ dishes coated overnight with collagen IV (Sigma, St. Louis, MO, USA). The non-adherent cells were then immediately rinsed off. The adherent cells were further cultured with fresh K-SFM in cell culture incubators at 37°C with 100% humidity and 5% CO_2_ in air. The medium was changed every other day. When the cells reached 60–70% confluence, those at passages 1 (P1) and P2 were digested with TrypLE Select (Invitrogen, CA, USA) for 10 min. Cells at P2 were used for identification and other experiments.

Cells at P2 were seeded on coverslips precoated with type IV collagen, fixed with 70% methanol in acetone, and blocked for 1 h with 1% bovine serum albumin (BSA, Sigma, St. Louis, MO, USA) at room temperature. Cells were incubated with mouse anti-human integrin β1 (1:200, Santa Cruz Biotechnology, CA, USA) and rabbit anti-human CK19 (Cytokeratin 19, 1:200, Sigma, Saint Louis, MO, USA) antibodies at 4°C overnight and then with TRITC-conjugated donkey anti-mouse secondary antibody (1:400, Invitrogen, CA, USA) and FITC-conjugated goat anti-rabbit secondary antibody (1:400, Invitrogen, CA, USA) for 1 h at room temperature. Finally, the stained cells were examined under a laser-scanning confocal fluorescence microscope (Leica, Munich, Germany).

### Transient cell transfection

Vectors encoding dominant-negative RhoA^(−/−)^ (RhoA T19N: threonine at residue 19 replaced with asparagine) or dominant positive RhoA^+/+^ (RhoA G14V: glycine at residue 14 replaced with valine) and the pGFP control plasmid were purchased from Fisher Scientific (Waltham, MA, USA) [27]. Specific PKN1 siRNA (sense 5'-CACAGUGUUUGAGAACUAUTT-3' and antisense 5'-GCUAGACGUGGGAAGAAAATT-3') and control siRNA were purchased from Thermo Fisher Scientific. In total, 60 nM siRNA, 1.5 μg/ml RhoA^(+/+)^(G14V), 1.5 μg/ml RhoA^(-/-)^(T19N) and 1.5 μg/ml pGFP control plasmid were transfected into huESCs using Lipofectamine 2000 (Invitrogen, California, USA), according to the manufacturer’s protocols. The cells were then stabilized in K-SFM for 24 h before further experimentation.

### Cell proliferation assay

ESCs were seeded in 96-well culture plates at 5,000 cells/well, allowed to stabilize for 48 h, and then treated with each agent. Following the addition of 10 μl of Cell Counting Kit-8 (CCK-8) (Dojindo, Kumamoto, Japan) solution to each well, the cells were incubated for an additional 4 h. Absorbance was measured at 450 nm with a microplate reader (SpectraMax 190; Molecular Devices). Growth curves were generated from the average values of five wells per group.

### DNA synthesis assay

Thymidine incorporation assays were performed as previously described [28]. ESCs were treated with each agent and pulsed with [^3^H]-thymidine (1 μCi/ml) (Life Sciences, Boston, MA) for 48 h. Cells were washed with PBS, incubated with 10% trichloroacetic acid, rinsed with a mixture of ethanol and diethylether (2:1), and dissolved in 0.5 N NaOH. The incorporation of [^3^H]-thymidine into cells was measured by a liquid scintillation counter.

### Western blotting

Phosphorylated PKN1 assays were conducted as previously described [29]. The stimulated cell lysates were resolved by 10% SDS-PAGE before transfer onto nitrocellulose membranes, which were blocked at room temperature with Tris-buffered saline (TBS) containing 3% BSA and then incubated overnight at 4°C with anti-PKN1, anti-Thr^774^ phosphorylated PKN1, anti-cyclin D1 and anti-glyceraldehyde 3-phosphate dehydrogenase (GAPDH) antibodies to detect target protein expression. Anti-Thr^774^ phosphorylated PKN1 primary antibodies were purchased from Cell Signaling Technology (Danvers, MA, USA), and the other antibodies were purchased from Sigma-Aldrich (St. Louis, MO, USA). The membranes were subsequently washed three times with TBS containing 0.1% Tween 20, incubated with horseradish peroxidase-conjugated secondary antibodies at room temperature for 1 h and washed again. Bands were visualized using enhanced chemiluminescence.

### RNA isolation and real-time PCR

For quantitative PCR analysis, total RNA was extracted using an RNeasy Mini Kit (QIAGEN), and cDNA was synthesized using a First Strand cDNA Synthesis Kit (TOYOBO). Real-time PCR analysis of human cDNA was performed with the 7500 Real-Time PCR System (Applied Biosystems) and SYBR Green. Expression values were normalized to GAPDH expression. The cyclin D1 (size, 101 bp) primer sequences were as follows: forward, 5'-CTACTACCGCCTCACACGCTT-3′; and reverse, 5'-GGCTTGACTCCAGGGCT-3′. The GAPDH (size, 87 bp) primer sequences were as follows: forward, 5'-TGCACCACCAACTGCTTAGC-3′; and reverse, 5'- GGCATGGACTGTGGTCATGAG-3′.

### Animals

C57BL/6 mice were provided by the Experimental Animal Department of the Third Military Medical University, China. All animal handling and experimental procedures were approved by the Committee on the Ethics of Animal Experiments of the Third Military Medical University. This study was carried out in strict accordance with the recommendations in the Guide for the Care and Use of Laboratory Animals of the National Institutes of Health. The animals were individually housed in plastic cages under standard conditions (temperature, 25°C; relative humidity, 50%; and circadian rhythm, 12 h). The animals were given standard autoclaved rodent chow and water ad libitum and were acclimated to the facility for 1 week before the experiment. All surgeries were performed under 0.1% sodium pentobarbital anesthesia, and all efforts were made to minimize suffering.

### ESC proliferation assay in a mouse model of burn injury

ESCs in mouse skin were labeled with BrdU [6,30], and a superficial partial-thickness burn model was generated as previously described [6]. Briefly, neonatal C57BL/6 mice were intraperitoneally injected with BrdU (50 mg/kg body weight, Sigma) twice daily for 3 days, beginning on day 3 after birth. Skin cells retaining BrdU after 7 weeks were identified as ESCs. After retaining BrdU for 7 weeks, the mice were anesthetized with an i.p. injection of 0.1% sodium pentobarbital at 10 μl per gram of body weight, followed by interscapular hair removal. Then, a metal plate (Shandong Academy of Medical Science, Jinan) with a diameter of 1.5 cm and a weight of 0.5 kg was used to induce superficial partial-thickness burns. The metal plate was heated to 65°C and placed evenly on the shaved mouse dorsum for 3 sec. The mice were individually housed in plastic cages under standard conditions.

The mice were then given an intraperitoneally injection of IdU (5-iodo-2-deoxyuridine, 100 mg/kg body weight, Sigma) and randomly divided into the following groups: U-46619 (RhoA agonist), C3 transferase (RhoA antagonist), U-46619+K-252a (PKN antagonist) and control (normal saline, NS). Immediately after being injured, the mice in each group were intraperitoneally injected (0.05 ml/g body weight) with U-46619 (10^−8^ mol/l, Sigma), C3 transferase (60 μg/l, Sigma), K-252a (5 μg/ml in NS, Santa Cruz) or NS (as control). Each group contained six mice. After 48 h, the mice were anesthetized with an i.p. injection of 0.1% sodium pentobarbital at 10 μl per gram of body weight and euthanized by cervical dislocation. The wounds were biopsied, fixed in paraformaldehyde and sectioned. The BrdU and IdU-labeled cells were detected using immunofluorescence. Six random fields in each section were imaged under a microscope at 400× magnification. The total number of BrdU^+^IdU^+^ cells in the re-epithelialization area in each field was counted using Image-Pro Plus (Media Cybernetics).

### Statistical analysis

The data are expressed as the mean ± SD of the indicated number of observations. In all instances where radioisotopes were used, background radioactivity was subtracted before quantifying radioactivity. Comparisons between two groups were performed using the unpaired, two-tailed Student's t-test. If necessary, one-way analysis of variance (ANOVA) was applied for comparisons among multiple groups. In all cases, P values less than 0.05 were considered significant.

## Results

### Establishment and characterization of human epidermal stem cells (huESCs)

**Fig 1:**
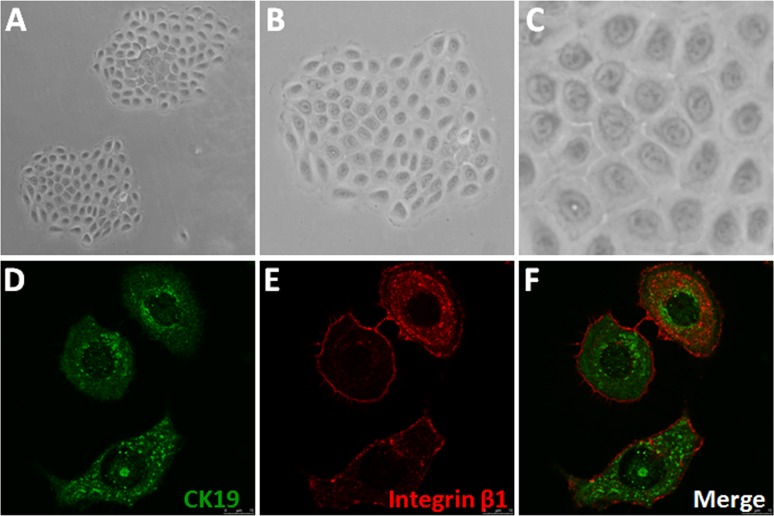
Characterization of isolated and cultured cells. (A) Cells behaved like clonogenic stem cells in vitro (×100). (B) Magnification of (A) (×200). (C) Magnification of (B) (×400). (D-F) The cells expressed integrin β1 and CK19 (immunofluorescence, ×1000).

As previously described [4], huESCs were enriched in our experiment based on collagen type IV adhesiveness. Approximately 50% of these rapidly adherent cells formed large colonies (Fig 1A, 1B and 1C) and grew to confluence within 2 weeks. Stem cells are defined by their uniquely slow cycling and high proliferative potential [31]. Basal keratinocytes expressing the cell surface proteins integrin β_1_ and CK19 exhibit many of the predicted characteristics of huESCs [32]. Integrin β_1_ and CK19 expression levels in the primary cultured P2 cells were examined using immunofluorescence staining. Almost all cells from the single colony expressed high levels of integrin β_1_ and CK19 (Fig 1D, 1E and 1F). The clonogenic capacity and expression of ESC markers indicated that the prepared cells were ESCs.

### RhoA effect on huESC proliferation

**Fig 2:**
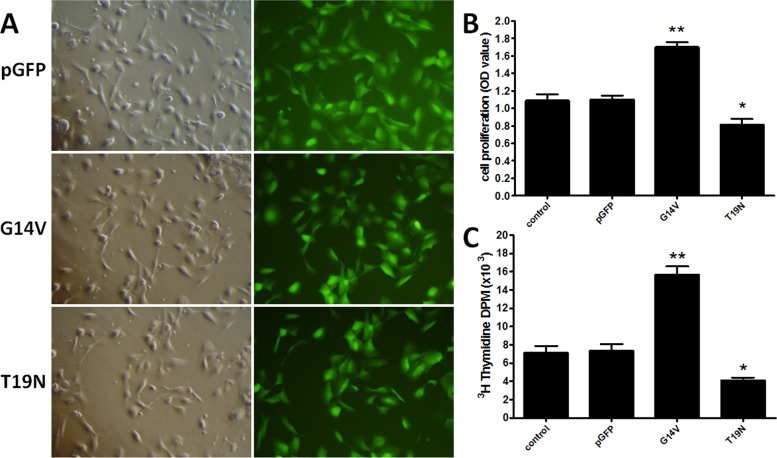
Effect of RhoA on huESC proliferation. (A) Transfection of RhoA^(+/+)^(G14V), RhoA^(-/-)^(T19N) or pGFP control plasmid (1.5 μg/ml each plasmid) into huESCs using Lipofectamine transfection reagent. (B) After transfection, cell proliferation was quantitated using a CCK-8 assay according to the manufacturer’s instructions. (C) DNA synthesis was quantified based on [^3^H]-thymidine uptake in disintegrations per minute (DPM). * P<0.05 versus control, **P<0.01 versus control. Data are shown as the mean ± SD of duplicate experiments on samples from three different donors (unpaired, two-tailed Student's t-test).

The RhoA^(+/+)^(G14V), RhoA^(-/-)^(T19N) and pGFP control plasmid transfection efficiencies were between 80–95% within 24 h of transfection as judged by GFP fluorescence (Fig 2A), indicating the successful transfection of each plasmid into huESCs. After transfection, a cell counting assay with CCK-8 and a DNA synthesis assay with [^3^H]-thymidine incorporation were used to assess cell proliferation. As shown in Fig 2B, compared with the control group, the G14V group showed a 56.11% increase in cell proliferation, and the T19N group showed a 25.13% decrease in proliferation. Moreover, compared with the control group, there was a 120.25% increase in DNA synthesis in the G14V group and a 42.66% decrease in the T19N group (Fig 2C). These results indicated a positive correlation between RhoA and huESC proliferation.

### Activation and phosphorylation of PKN1 by RhoA in huESCs

**Fig 3:**
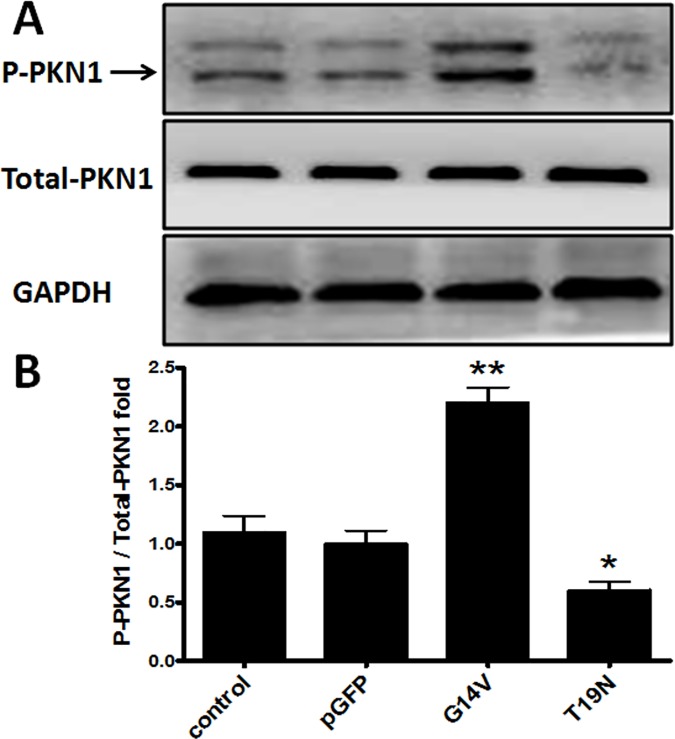
RhoA induces PKN1 activation and phosphorylation. HuESCs were transfected for 48 h with RhoA^(+/+)^(G14V), RhoA^(-/-)^(T19N) or pGFP control plasmid (1.5 μg/ml each plasmid) using Lipofectamine transfection reagent. (A) Cell lysates were subjected to Western blot assays with anti-Thr^774^ phosphorylated (p-)PKN1, anti-total PKN1 and anti-GAPDH antibodies. (B) Thr^774^ p-PKN1/total-PKN1 was determined after densitometric analysis. *P<0.05 versus control, **P<0.01 versus control. The data are presented as the mean ± SD (error bars) of at least three separate experiments (unpaired, two-tailed Student's t-test).

As shown in Fig 3, the total protein levels of PKN1 did not differ among huESCs transfected with G14V, T19N, pGFP plasmid or control huESCs. Compared to the control group, PKN1 activation increased by 120.25% in the G14V group (P<0.01), decreased by 39.33% in the T19N group (P<0.05), and was not significantly different in the pGFP group. These results indicate that PKN1 is activated by RhoA.

### Specific PKN1 siRNA-mediated knock-down inhibits the RhoA enhancement of huESC proliferation

**Fig 4:**
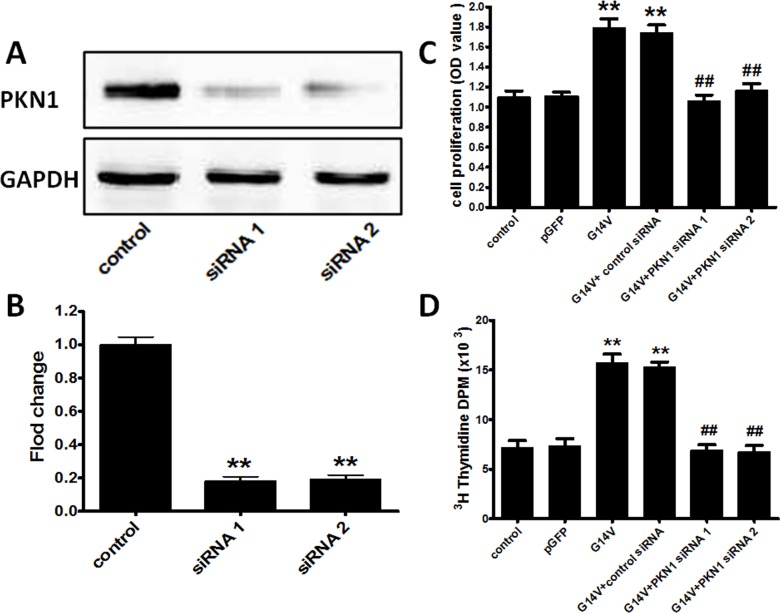
RhoA regulates huESC proliferation via PKN1 signaling: evidence from siRNA-mediated PKN1 knock-down assays. (A) A representative Western blot demonstrating the effects of two distinct PKN1 siRNAs. (B) Quantification of the Western blots in (A); **P<0.001 versus control siRNA. After Lipofectamine-mediated transfection of 1.5 μg/ml G14V plasmid, 1.5 μg/ml pGFP control plasmid, and 60 nM specific PKN1 siRNA into huESCs, a cell counting assay with CCK-8 (C) and a DNA synthesis assay with [^3^H]-thymidine incorporation (D) were used to assess cell proliferation at a 48-h timepoint. The data are presented as the mean ± SD of three independent experiments, each performed in duplicate (n = 3). The unpaired, two-tailed Student's t-test was used to assess significance: **P<0.001 versus control, ^##^P<0.001 versus G14V + control siRNA.

As shown in Fig 4A, the knock-down efficiencies of the two distinct PKN1 siRNAs were 81.63±5.86% and 80.96±6.63%. After transfection of G14V, pGFP control plasmid, and PKN1 siRNA, a cell counting assay with CCK-8 (Fig 4C) and a DNA synthesis assay with [^3^H]-thymidine incorporation (Fig 4D) were used to assess cell proliferation. As shown in Fig 4C, compared with the control group, the G14V mutant significantly increased cell proliferation, and this effect was abolished by PKN1 siRNA. The same effects were revealed by the DNA synthesis assay (Fig 4D). These results indicate that the ability of RhoA to promote huESC proliferation may be mediated by PKN1 signaling.

### RhoA regulates cyclin D1 protein and mRNA expression in huESCs through PKN1

**Fig 5:**
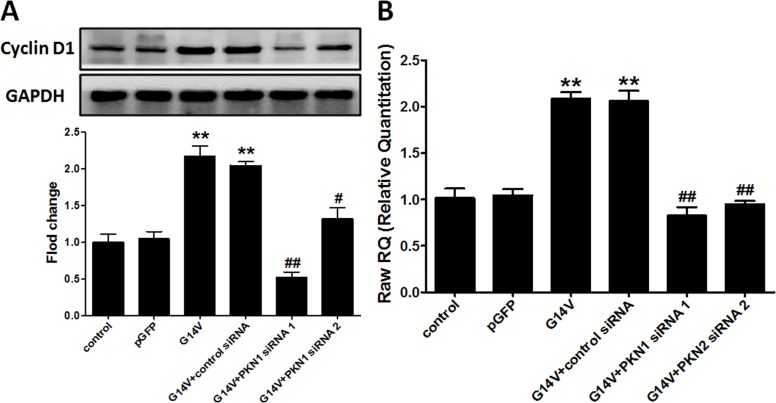
RhoA regulates cyclin D1 expression through PKN1. After Lipofectamine-mediated transfection of huESCs with 1.5 μg/ml G14V plasmid, 1.5 μg/ml pGFP control plasmid, and 60 nM specific PKN1 siRNA for 48 h, cyclin D1 protein (Western blot, A) and mRNA expression (real-time PCR, B) was detected. The data are presented as the mean ± SD of three independent experiments; an unpaired, two-tailed Student's t-test was used to assess significance: **P<0.01 versus control, ^#^P<0.05 versus G14V + control siRNA, ^##^P<0.001 versus G14V + control siRNA.

As shown in Fig 5A, cyclin D1 protein expression was higher in the G14V group than in the control group (P<0.01), and this effect was blocked by PKN1 siRNA to different degrees. There were no significant differences between the pGFP group and the control group and between the G14V group and the G14V + control siRNA group. Based on real-time PCR assays, cyclin D1 mRNA expression was significantly augmented in cells transfected with G14V compared to control cells (P<0.01), and the effects could be blocked by PKN1 siRNA (Fig 5B). These results show that RhoA regulates the transcription of cyclin D1 via PKN1.

### The effect of RhoA on ESC proliferation via PKN in vivo

**Fig 6:**
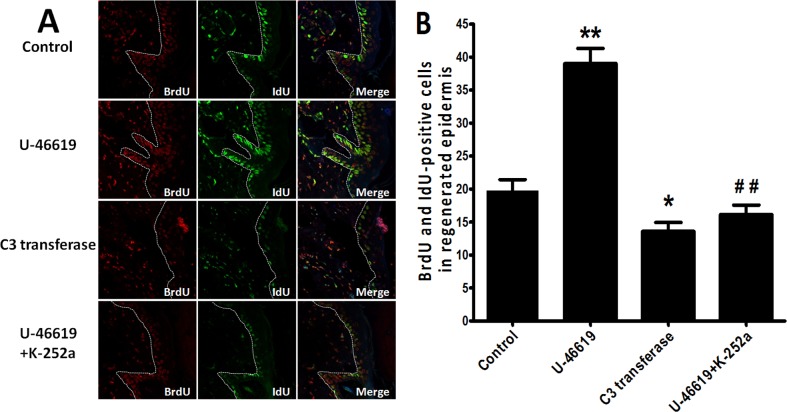
Immunohistochemical analysis of BrdU^+^IdU^+^-labeled cells. After BrdU labeling of ESCs and IdU incorporation, immunofluorescence staining for BrdU and IdU was performed in mice treated with normal saline, U-46619, C3 transferase or U-46619+K-252a (A). 400× magnification. Double-positive cells are visible in the regenerated epidermis. Representative images of well-formed, BrdU and IdU-positive cells in the regenerated epidermis are shown at the same magnification (A). (B) Data analysis was performed using ImagePro Plus. Columns represent the mean double-positive cell count in the regenerated epidermis. The data are presented as the mean ± SD of three independent experiments; an unpaired, two-tailed Student's t-test were used to assess significance: *P<0.05 versus control group, **P<0.01 versus control group, ^##^P<0.01 versus U-46619 group.

To examine the effect of RhoA on ESC proliferation in vivo, BrdU-labeled ESCs and superficial second-degree burns were established in vivo as previously described [6]. After IdU incorporation and treatment with reagents for 48 h, the wounds were biopsied and sectioned. BrdU-positive cells were present in the regenerated epidermis (Fig 6A and 6B). The total number of double BrdU and IdU-positive cells in the re-epithelialization area in each field was counted (Fig 6B). In the in vivo ESC proliferation experiments, the number of BrdU^+^IdU^+^ positive cells in the regenerated epidermis was significantly different in the U-46619 group compared with the control group (P<0.01) and was significantly decreased in the C3 transferase group compared to the control group (P<0.05). Moreover, the PKN inhibitor K-252a abolished the effect of U-46619 on the number of BrdU-positive cells in the regenerated epidermis. The data show that RhoA promotes the proliferation of ESCs through PKN.

## Discussion

In this study, we are the first to demonstrate that RhoA is a novel target for the proliferation of ESCs, which were enriched by rapid adhesion to collagen IV (Fig 1), and that the RhoA-mediated up-regulation of cyclin D1 via PKN1 activation is a new mechanism for ESC proliferation, suggesting that RhoA may participate in wound healing.

Rho GTPases play a central role in cell migration, though the relative contribution of each Rho GTPase depends on the environment and cell type [33]. Furthermore, Rho GTPases have been reported to regulate proliferation and the cell cycle in cancer cells [34] and nerve cells [35]. Recently, the Rho GTPase Rac1 was reported to regulate ESC proliferation and to determine ESC fate by controlling their exit from the stem cell compartment [4]. Furthermore, Rho GTPase was reported to be involved in nitric oxide-induced ESC migration. We found that in contrast to a dominant-negative RhoA mutant (T19N), an active RhoA mutant (G14V) promotes ESC proliferation and cell DNA synthesis in vitro (Fig 2B and 2C). Additionally, in contrast to the RhoA antagonist C3 transferase, the RhoA agonist U-46619 promotes ESC proliferation during wound healing (Fig 6), suggesting that RhoA plays an important role in ESC proliferation. ESC biological activities, such as proliferation and migration, have functions in wound healing. For example, Horii et al. [36] reported a link between RhoA and intestinal epithelium wound healing. In vivo, ESCs participate in wound healing via a continuous process of de-adhesion, migration, differentiation and proliferation. In this study, we attempted to detect ESC proliferation in vivo by designing a model based on the BrdU pulse-chase method reported by Taylor et al. [30] combined with IdU incorporation and their observation that double BrdU and IdU-positive cells must undergo proliferation in the re-epithelialization area. Moreover, as we previously reported, RhoA is involved in ESC migration under nitric oxide treatment [6]. Rho GTPases play important roles in cell migration and spreading, whereby the effect of RhoA in the wound healing process involves the promotion of ESC proliferation and migration.

There is no question based on the literature that cyclin D1 is a critical modulator of the G1/S cell cycle transition [24,37]. Cyclin D1 is a key regulator of cell proliferation, and its expression is subject to both transcriptional and post-transcriptional regulation. Cyclin D1 protein and mRNA expression levels were up-regulated by an active RhoA mutant (G14V), and these effects were inhibited by siRNA-mediated knock-down of PKN1 (Fig 5A and 5B). Moreover, the ability of RhoA to promote ESC proliferation and DNA synthesis was interrupted by PKN1 siRNA (Fig 4C and 4D), and the PKN inhibitor K-252a abolished the effect of U-46619 on BrdU-positive cells in the regenerated epidermis during wound healing (Fig 6). Simultaneously, PKN1 was activated by RhoA (Fig 3A and 3B). All these findings indicate that RhoA up-regulated cyclin D1 through PKN1 to promote cell proliferation and suggest a new mechanism by which RhoA regulates cell proliferation. It has been reported that PKN1, as the downstream effector of RhoA, regulates cytoskeleton organization [20,38]. Furthermore, PKN1 can utilize coiled-coil motifs to interact with a few residues within the RhoA switch regions [39,40]. The relationship between PKN1 and cyclin D1 is nebulous and rarely reported. Singh et al. [23] reported that the depletion of cyclin D1 levels abolished monocyte chemotactic protein-1 (MCP-1)-induced PKN1 phosphorylation in smooth muscle cell migration and proliferation. Our study found that cyclin D1 is a downstream regulator of PKN1, which can regulate the transcription of cyclin D1.

In conclusion, this study shows that RhoA plays an important role in ESC proliferation in vitro and in vivo and is likely involved in wound healing. Moreover, we found a new mechanism by which RhoA regulates ESC proliferation via activation of the PKN1-cyclin D1 signaling pathway in vitro and in vivo, suggesting that RhoA may serve as a new therapeutic target for wound healing.
